# Umbilical cord blood-derived microglia-like cells to model COVID-19 exposure

**DOI:** 10.1038/s41398-021-01287-w

**Published:** 2021-03-19

**Authors:** Steven D. Sheridan, Jessica M. Thanos, Rose M. De Guzman, Liam T. McCrea, Joy E. Horng, Ting Fu, Carl M. Sellgren, Roy H. Perlis, Andrea G. Edlow

**Affiliations:** 1 Department of Psychiatry, Harvard Medical School, Boston, MA USA; 2 Center for Genomic Medicine and Department of Psychiatry, Massachusetts General Hospital, Boston, MA USA; 3 Obstetrics, Gynecology, and Reproductive Biology, Harvard Medical School, Boston, MA USA; 4 Vincent Center for Reproductive Biology, Massachusetts General Hospital, Boston, MA USA; 5 Department of Physiology and Pharmacology, Karolinska Institutet, Stockholm, Sweden

**Keywords:** Molecular neuroscience, Stem cells

## Abstract

Microglia, the resident brain immune cells, play a critical role in normal brain development, and are impacted by the intrauterine environment, including maternal immune activation and inflammatory exposures. The COVID-19 pandemic presents a potential developmental immune challenge to the fetal brain, in the setting of maternal SARS-CoV-2 infection with its attendant potential for cytokine production and, in severe cases, cytokine storming. There is currently no biomarker or model for in utero microglial priming and function that might aid in identifying the neonates and children most vulnerable to neurodevelopmental morbidity, as microglia remain inaccessible in fetal life and after birth. This study aimed to generate patient-derived microglial-like cell models unique to each neonate from reprogrammed umbilical cord blood mononuclear cells, adapting and extending a novel methodology previously validated for adult peripheral blood mononuclear cells. We demonstrate that umbilical cord blood mononuclear cells can be used to create microglial-like cell models morphologically and functionally similar to microglia observed in vivo. We illustrate the application of this approach by generating microglia from cells exposed and unexposed to maternal SARS-CoV-2 infection. Our ability to create personalized neonatal models of fetal brain immune programming enables non-invasive insights into fetal brain development and potential childhood neurodevelopmental vulnerabilities for a range of maternal exposures, including COVID-19.

## Introduction

Maternal immune activation can result from exposures ranging from metabolic conditions to stress and infection, with potential in utero consequences to the developing fetus^1–6^. In particular, epidemiologic studies strongly suggest that maternal viral and bacterial infections in pregnancy may be associated with adverse neurodevelopmental outcomes in offspring, particularly autism spectrum disorders, schizophrenia, and cerebral palsy, but potentially including mood and anxiety disorders as well^1–3,7–9^. For instance, individuals who were fetuses during the 1957 influenza pandemic had a significantly increased risk for being hospitalized for schizophrenia as an adult^10^. The magnitude of these effects varies, but their consistency is difficult to ignore. Although mechanisms underlying neurodevelopmental morbidity in offspring remain unclear, microglial priming toward a pro-inflammatory phenotype with consequent altered synaptic pruning has been suggested as a candidate mechanism^11–17^.

Microglia, brain-resident tissue macrophages, play a key role in normal neurodevelopment by modulating synaptic pruning, neurogenesis, phagocytosis of apoptotic cells, and regulation of synaptic plasticity^18–21^. Fetal yolk sac-derived macrophages are the progenitors for the permanent pool of brain microglia throughout an individual’s lifetime^22–25^. As such, inappropriate fetal microglial priming (“trained immunity”^26^) due to in utero immune activation may have lifelong neurodevelopmental consequences. The central role of mononuclear cells, including macrophages, in COVID-19 pathogenesis^27^ suggests that the potential risk to exposed fetal microglia requires investigation.

We have previously developed and validated adult patient-specific models of microglia-mediated pruning by reprogramming induced microglial cells from human peripheral blood mononuclear cells (PBMCs), and assaying them with isolated synapses (synaptosomes) derived from neural cultures differentiated from induced pluripotent stem cells (iPSCs)^28,29^. We demonstrated robust evidence of abnormalities in microglia and synaptosomes from individuals with schizophrenia, shown to be complement-dependent through a C3 receptor neutralizing antibody and rescued in a dose-responsive fashion with a small molecule probe^28^. Other groups have similarly applied in vitro synaptic pruning assays to provide insight into the pathogenesis of autism spectrum disorder and neurodegenerative disorders^30–32^.

In this study, we investigated whether our validated reprogramming methods for adult PBMCs could be successfully adapted and applied to umbilical cord blood-derived mononuclear cells (CB-MNCs) from both SARS-CoV-2 infected and uninfected pregnancies to create personalized models of fetal brain microglia. Such models could have a wide range of application in investigating effects of in utero exposure on neurodevelopment. To illustrate this application, we demonstrate successful induction of microglia-like cells (CB-iMGs) from CB-MNCs from both infected and uninfected pregnancies.

## Materials/subjects and methods

### Ethical statement

This study was approved by the Partners Institutional Review Board. Informed consent was obtained from all participants.

### Isolation and preparation of mononuclear cells from umbilical cord blood (CB-MNCs)

Umbilical cord blood was collected at the time of delivery into EDTA tubes for plasma and CB-MNC isolation. After spinning at 1000*g* for 10 min to separate plasma, samples were processed for CB-MNC isolation using a Ficoll density gradient^33^. Briefly, blood was transferred into a 50 mL conical tube and then diluted to 1:1 ratio with Hanks’ balanced salt solution without calcium or magnesium (HBSS minus). This diluted blood was then gently layered on top of Ficoll at 2:1 ratio (two volumes of blood diluted with HBSS minus to one volume Ficoll). The conical tube was then centrifuged at 1000*g* for 30 min at room temperature with brake inactivated to allow layering of cellular components. The cloudy ring below the plasma and above the Ficoll (i.e. the CB-MNC layer) was collected and placed in a new 15 mL conical tube, with HBSS minus added to bring the volume to 15 mL. This tube was then centrifuged at 330*g* for 10 min with high brake. The supernatant was removed and the CB-MNC pellet was washed with HBSS minus and resuspended in 10 mL HBSS minus for counting. Cells were counted on a hemocytometer in a 1:10 dilution of trypan blue. Cells were frozen in freezing medium consisting of RPMI 1640 Medium with 1% penicillin–streptomycin, l-glutamine, 1% sodium pyruvate, 1% non-essential amino acids, 20% fetal bovine serum (FBS), and 10% DMSO at 5–10 million cells/vial, placed in a chilled Mr. Frosty, then into −80 °C. The following day, CB-MNC cryovials were transferred to liquid nitrogen for long-term storage. Isolated cryopreserved adult peripheral blood mononuclear cells were obtained from a single healthy control donor by Vitrologic (https://vitrologic.com) cat# MNC-300.

### Derivation of induced microglia-like cells from CB-MNCs by direct cytokine reprogramming

iMGs were derived using previously described methods^28,29^ while CB-iMGs were derived from CB-MNCs with modifications as noted. Briefly, cryopreserved PBMCs or CB-MNCs were rapidly thawed at 37 °C, diluted into media consisting of RPMI 1640 with 10% FBS and 1% penicillin/streptomycin. The cell suspension was centrifuged at 300*g* for 5 min at room temperature, with the brake off. After aspirating the supernatant, the cell pellet was resuspended in media, counted, and plated on Geltrex-coated 24-well plates at 1 × 10^6^ cells per 0.5 mL per well. After cells were incubated at 37 °C for 24 h, the media was completely replaced with RPMI 1640 with GlutaMAX, 1% penicillin–streptomycin, 100 ng/mL of human recombinant IL-34 (Peprotech), and 10 ng/mL of GM-CSF (Peprotech). Media was replaced on day 13 after the initial cytokine reprogramming, and real-time live cell imaging or immunocytochemistry was performed on day 14.

### iPSC generation and neural differentiation for synaptosome isolation

iPSCs were reprogrammed from fibroblasts and used to derive neural progenitor cells, which were differentiated into neural cultures, as previously described^28,29^. In brief, adult human fibroblasts were reprogrammed to iPSCs using non-integrating synthetic RNA pluripotency factors, expanded and cryopreserved by Cellular Reprogramming, Inc. (https://www.cellular-reprogramming.com). To initiate neural progenitor induction, iPSCs were cultured feeder-free in E8 medium (Gibco) on Geltrex-coated six-well plates and passaged using 50 mM EDTA and trituration with ROCK inhibitor (10 mM Thiazovivin; Stemgent). iPSCs were further purified using magnetic-activated cell sorting with Tra-1-60 microbeads (Miltenyi Biotec) on LS columns as described by vendor. Neural progenitor cells (NPCs) were derived from these iPSCs using neurobasal medium (Thermo Fisher Scientific) with 1× Neural Induction Supplement (Thermo Fisher Scientific), expanded using a neural expansion medium, and purified by double sorting using MACS against CD271 and CD133. NPCs were immunostained for markers, including Nestin, SOX1, SOX2, and Pax-6. Validated NPCs were seeded for neural differentiation on Geltrex-coated T1000 five-layer cell culture flasks (Millipore Sigma # PFHYS1008) and grown in neuronal differentiation medium (Neurobasal media (Gibco # 21103049) supplemented with 1× each (N2 supplement (Stemcell Technologies SCT # 7156), B27 supplement without Vitamin A (Gibco # 12587010), non-essential amino acids (NEAA Gibco # 11140050), penn/strep), 1 μM ascorbic acid, 10 ng/mL BDNF and GDNF (Peprotech), and 1 μg/mL mouse laminin (Sigma # L2020) for 8 weeks.

### Synaptosome isolation by sucrose gradient

Synaptosome isolation by sucrose gradient was adapted for iPSC-derived differentiated neural cultures from previously described protocols^34–36^. First, media was aspirated from flasks and cells were washed or scraped with 1× gradient buffer (ice-cold 0.32 M sucrose, 600 mg/L Tris, 1 mM NaH_3_CO_3_, 1 mM EDTA, pH 7.4 with added HALT protease inhibitor—Thermo Fisher # 78442), homogenized using a dounce homogenizer and centrifuged at 700*g* for 10 min at 4 °C. The pellet was resuspended in 10 mL of 1× gradient buffer (transferred to a 30 mL and centrifuged at 15,000*g* for 15 min at 4 °C. The second pellet was resuspended in 1× gradient buffer and slowly added on top of a sucrose gradient with 1× gradient buffer containing 1.2 M (bottom) and 0.85 M (middle) sucrose layers. The gradient and cell mixture was centrifuged at 26,500 r.p.m. (~80,000*g*) for 2 h at 4 °C, with the brake set to “slow” so as not to disrupt the final bands. The mixtures were handled carefully and bands were inspected to confirm successful fractionation. The synaptosome band (in between 0.85 and 1.2 M sucrose) was removed with a 5-mL syringe and 19Gx1 ½″ needle and centrifuged at 20,000*g* for 20 min at 4 °C. The final pellet was resuspended in an appropriate volume of 1× gradient buffer with 1 mg/mL bovine serum albumin (BSA) with protease and phosphatase inhibitors, aliquoted and slowly frozen at −80 °C. Protein concentration was measured by BCA, and synaptosomes were further analyzed by transmission electron microscopy for heterogeneity of size and morphology, and enrichment of pre-synaptic (synapsin, SNAP-25) and post-synaptic (PSD-95) markers was determined by western blot analysis as previously described^28,29^.

### Real-time live cell imaging of synaptosome phagocytosis by CB-iMGs

Real-time live cell imaging of CB-iMGs was performed as previously described^28,29^. Briefly, cells were imaged on the IncuCyte ZOOM live imaging system (Essen Biosciences) while incubated at 37 °C with 5% CO_2_. Synaptosomes were sonicated and labeled with pHrodo Red SE (Thermo Fisher Scientific) and added to CB-iMGs at 15 µg total protein per well in 24-well plates. Phase contrast and red fluorescence channel images were taken at a resolution of 0.61 µm per pixel every 45 min for a total of 315 min. Images were exported as 16-bit grayscale files and analyzed using CellProfiler^37^ to quantify cells and phagocytized particles. CellProfiler pipeline description are included in Supplementary Materials.

### Immunocytochemistry and confocal microscopy

iMGs were fixed with 4% paraformaldehyde in phosphate-buffered saline (PBS) for 15 min at 4 °C, washed twice with PBS, blocked for 1 h with 5% FBS and 0.3% Triton-X (Sigma Aldrich) in PBS, and then washed three times with 1% FBS in PBS. Cells were incubated with primary antibodies in 5% FBS and 0.1% Triton-X overnight at 4 °C (Anti-IBA1, 1:500; Abcam #ab5076; Anti-CX3CR1, 1:100, Abcam ab8021; Anti-PU.1, 1:11,000, Abcam #ab183327, and Anti-P2RY12, 1:100, Alomone Labs). Cells were then washed twice with 1% FBS in PBS and incubated in secondary antibodies (1:500) and Hoechst 33342 (1:5000) in 5% FBS and 0.1% Triton-X in PBS for 45 min at 4 °C, light-protected. Cells were washed twice and imaged using the IN Cell Analyzer 6000 (Cytiva). CellProfiler pipeline description used for quantification of marker immunopositive cell percentages are included in Supplementary Materials.

#### Quantitation of microglial marker gene expression by qRT-PCR

Total RNA was extracted using the RNeasy Plus Micro Kit (Qiagen # 74034) and treated with dsDNAse (Thermo Scientific # EN0771) for 10 min at 37 °C followed by heat inactivation for 5 min at 55 °C in the presence of 10 mM DTT. Forty-five nanograms of purified RNA was used for cDNA synthesis using the Superscript III First-Strand Synthesis System (Invitrogen # 18080051). Generated cDNA was diluted 1:3 and 3 μL were loaded into the quantitative PCR reaction containing TaqMan Fast Advanced Master Mix (Applied Biosystems # 4444556) and the TaqMan Gene Expression Assays (1×) for both the gene of interest and the housekeeping gene (Applied Biosystems; IBA1: Hs00610419_g1, PU.1: Hs02786711_m1, P2RY12: Hs00224470_m1, TMEM119: Hs01938722_u1, RPLP0: Hs00420895_gH). All reactions were run in technical triplicates in a 10 μL reaction volume in a 384-well plate with a Roche LightCycler 480 machine as follows: 50 °C for 2 min, 95 °C for 20 s, then 40 cycles of 95 °C for 1 s and 60 °C for 20 s. Ct values over 35 were set to the baseline Ct of 35 given inconsistent detection that occurs with low transcript levels. Relative quantification of gene expression was normalized to the endogenous housekeeping gene (RPLP0) then to the experimental negative control (PBMCs, CB-MNCs, respectively).

## Results

### Generating and characterizing human microglia-like cells from umbilical cord blood-derived mononuclear cells (CB-iMGs)

**Fig. 1:**
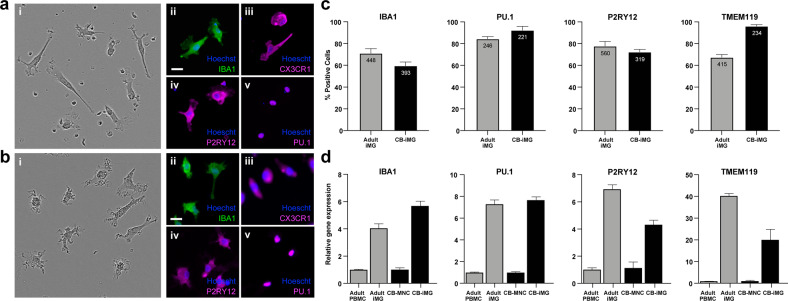
Characterization of monocyte-derived induced microglia-like cells (iMGs) by direct cytokine reprogramming. **a** Umbilical cord monocyte-derived CB-iMGs. **b** Adult PBMC-derived iMGs. (i) Morphology by phase contrast; immunostained images of iMG cells stained with nuclei (Hoechst) and indicated microglial markers (ii) IBA1, (iii) CX3CR1, (iv) P2RY12, and (v) PU.1. Scale bar: 30 μm. **c** Quantitation of positively immunostained cells as a percentage of total cells (nuclei) for indicated microglial markers. Bars indicate mean of indicated no. of cells measured, error bars indicating SEM. **d** Quantitation of fold increase in gene expression of indicated microglial markers normalized to input (PBMCs and CB-MNCs, respectively) after iMG reprogramming, error bars indicating SEM (*n* = 3 measurements).

We adapted previously reported methods^28,29^ for generating iMGs from adult-derived PBMCs to reprogram umbilical CB-MNCs from neonates of SARS-CoV-2 negative (*n* = 4) and SARS-CoV-2 positive (*n* = 2) mothers, delivered between 7 June 2020 and 6 July 2020. After 2 weeks of cytokine exposure, analogous to PBMC-derived iMGs, CB-iMGs displayed typical ramified microglial morphology (Fig. 1a, i) and stained positive for canonical microglial markers IBA1, CX3CR1, PU.1, and P2RY12 (Fig. 1a, ii–v), demonstrating that iMGs can be generated from umbilical cord blood-derived CB-MNCs. These cells exhibit morphologic and features and markers of cell identity comparable to those we have demonstrated for adult blood-derived PBMCs (Fig. 1b, i–v). Quantitation of immunopositive cells for microglial markers (Fig. 1c) and upregulation of canonical microglial genes (Fig. 1d) indicate comparable efficiency of iMG transdifferentiation by PBMCs and CB-MNCs.

### CB-iMGs demonstrate capacity to engulf isolated synaptic material in an in vitro model of synaptic pruning

**Fig. 2:**
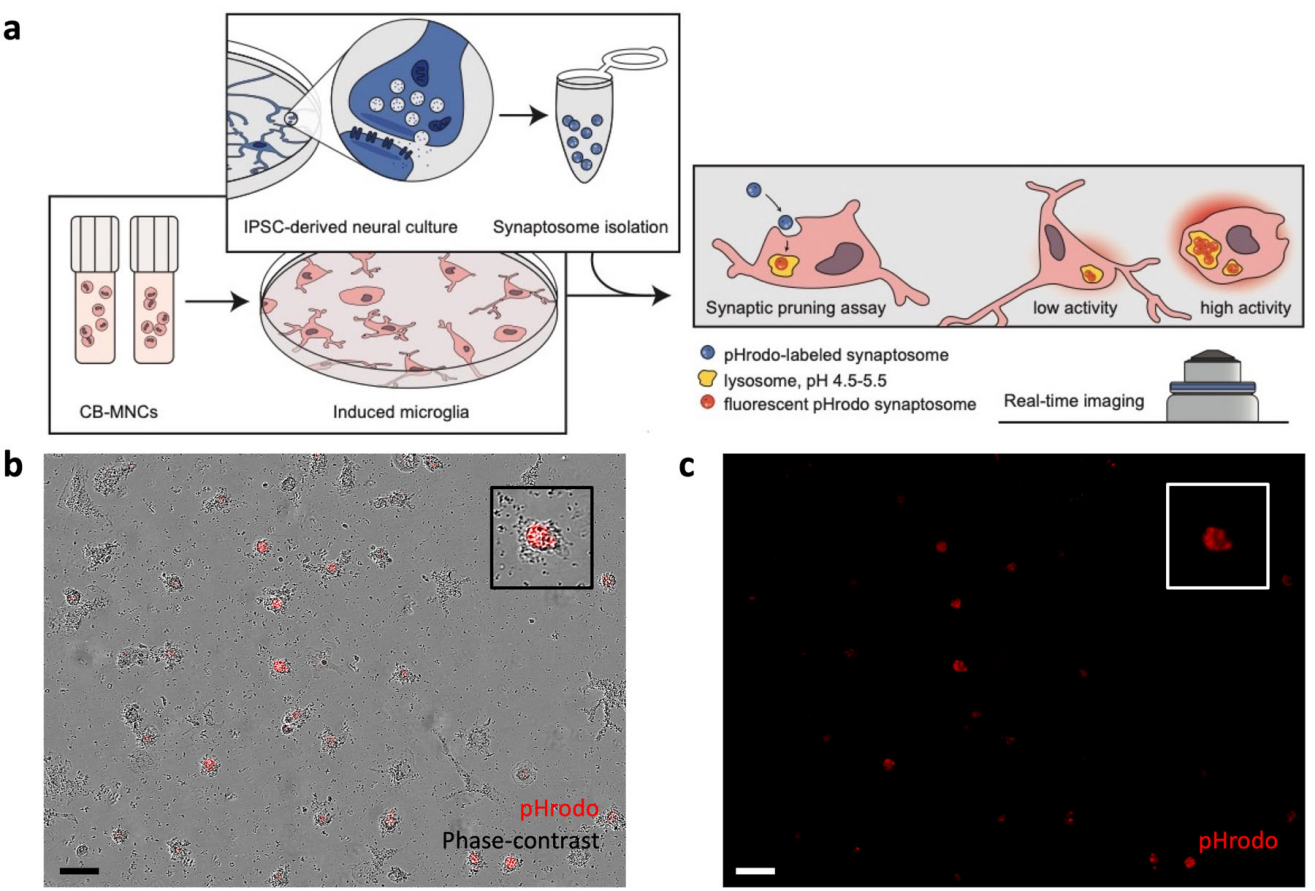
Characterizing synaptosome engulfment by CB-iMGs in an in vitro model of synaptic pruning. **a** Overall schematic of pHrodo-labeled quantitative synaptosome phagocytosis assay by CB-iMGs. **b** Representative live real-time images in phase contrast/red fluorescence overlay mode showing cellular uptake and **c** red fluorescence channel alone of pHrodo (red)-labeled synaptosomes uptake at the end of the phagocytosis assay (315 min). Scale bar: 60 μm (boxes show magnified view of engulfing CB-iMG).

We next characterized CB-iMG function in a model of synaptic pruning using highly purified isolated nerve terminals (synaptosomes), allowing quantitation of synaptic engulfment in vitro with greater signal-to-noise than intact neural cultures^28,29^. Figure 2a illustrates the real-time imaging-based phagocytosis assay workflow with CB-iMG cultures upon addition of iPSC-derived neural culture purified synaptosomes labeled with pHrodo Red SE, a pH-sensitive dye that fluoresces upon localization to lysosomes post-engulfment. Engulfment of synaptosomes can be robustly quantified in real-time live imaging (Fig. 2b, c) as well as by endpoint confocal microscopy of fixed immunostained cells^28,29^.

**Fig. 3:**
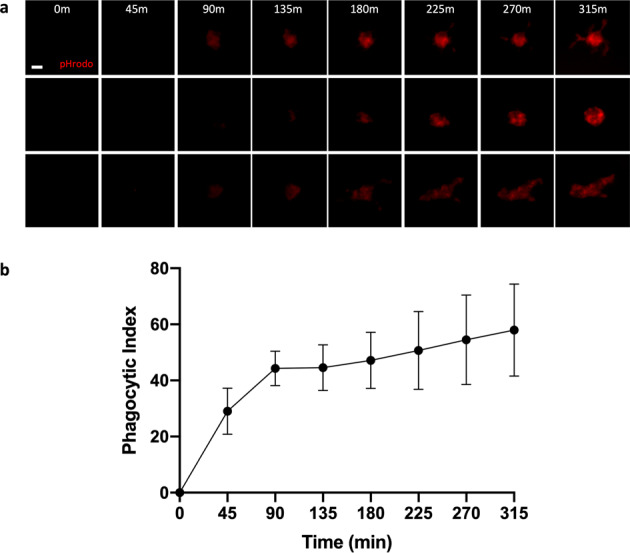
Quantifying synaptosome engulfment by CB-iMGs using live real-time imaging. **a** Representative pHrodo (red)-labeled synaptosome engulfment in iMG cells during live real-time imaging used for quantification Scale bar: 20 μm. **b** Quantification of labeled synaptosome uptake by CB-iMGs cells during live imaging. The phagocytic index represents the mean pHrodo+ area per iMG cell over *N* = 8 fields per well × 3 wells per line and *N* = 4 separate healthy control CB-IMG line derivations. Error bars represent SEM.

To characterize kinetics of phagocytosis over time, pHrodo-labeled synaptosomes were added to the CB-iMG cultures and engulfment was visualized every 45 min for approximately 5 h using real-time live fluorescence imaging (Fig. 3a). Phagocytotic index was determined at each time point by measuring pHrodo area per cell (Fig. 3b) using CellProfiler as described in “Methods” and Supplementary Information. CB-iMGs demonstrate robust phagocytosis of synaptosomes, with a time course of synaptosome engulfment rising over time qualitatively similar to that observed with adult PBMC-derived iMGs^28,29^.

### CB-IMGs derived from CB-MNCs exposed to maternal SARS-CoV-2 infection

**Fig. 4:**
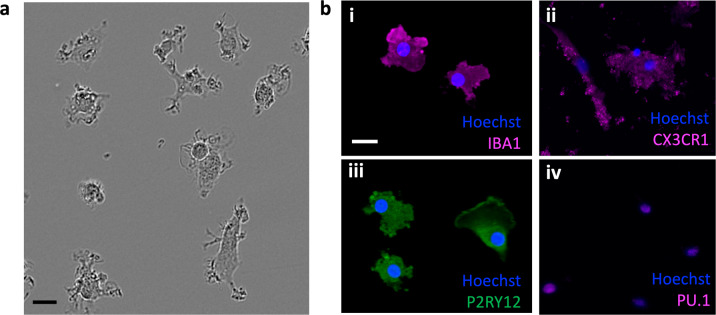
Phenotypic characterization of CB-iMGs derived from CB-MNCs exposed to maternal SARS-CoV-2 infection. **a** Phase contrast ramified morphology of patient-derived CB-iMGs. Scale bar: 30 μm. **b** Immunostained images of exposed CB-iMG cells with nuclei (Hoechst) and indicated microglial markers (i) IBA1, (ii) CX3CR1, (iii) P2RY12, and (iv) PU.1. Scale bar: 25 μm.

**Fig. 5:**
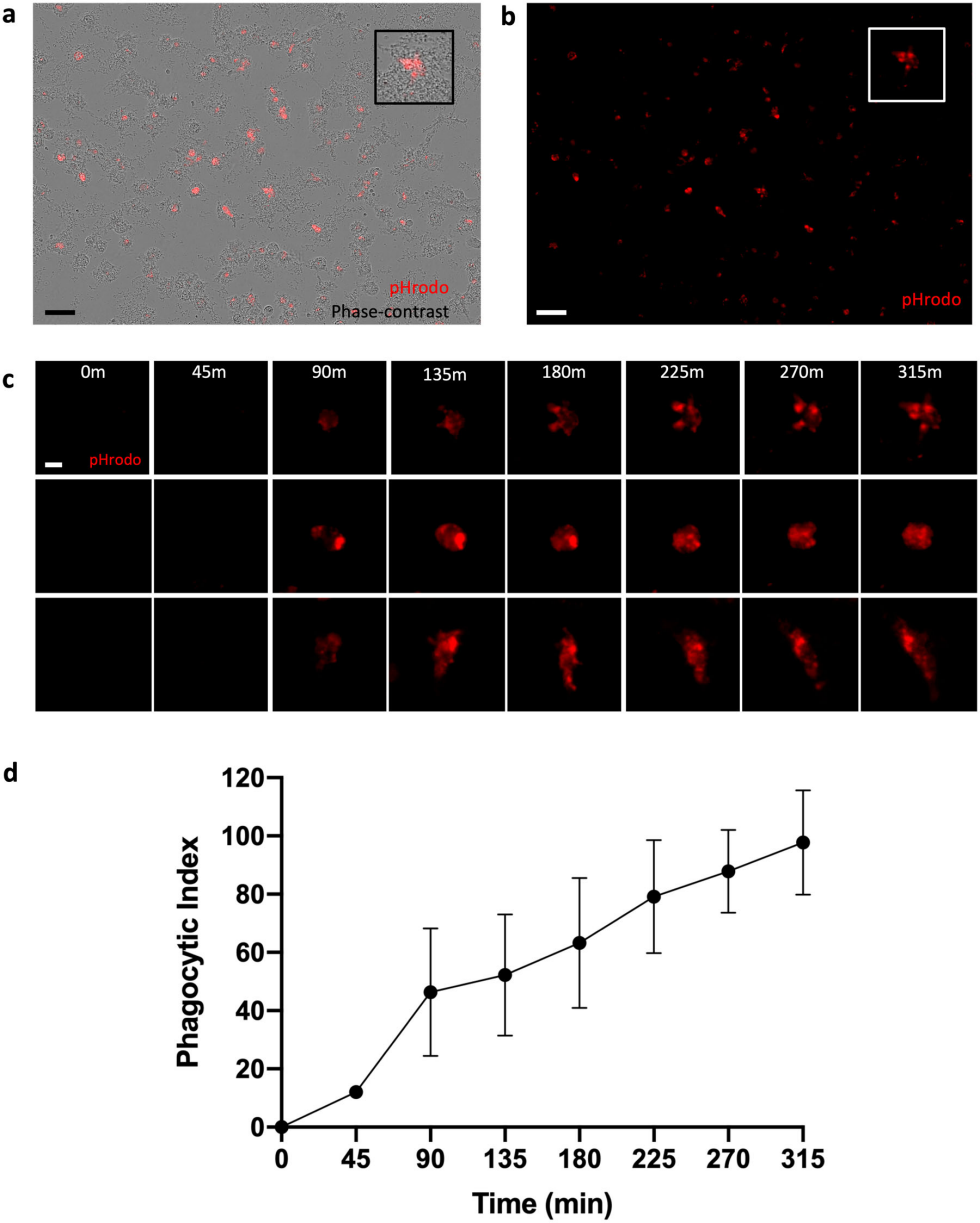
Functional characterization of synaptosome engulfment by CB-iMGs derived from CB-MNCs exposed to maternal SARS-CoV-2 infection. **a** Representative live real-time images in phase contrast/red fluorescence overlay mode showing cellular uptake and **b** red fluorescence channel alone of pHrodo (red)-labeled synaptosomes uptake at the end of the phagocytosis assay (315 min). Scale bar: 60 μm (boxes show the magnified view of engulfing CB-iMG). **c** Representative pHrodo (red)-labeled synaptosome engulfment in exposed CB-iMGs during live real-time imaging used for quantification. Scale bar: 20 μm. **d** Quantification of labeled synaptosome uptake by exposed CB-iMGs cells during live imaging. The phagocytic index represents the mean pHrodo+ area per iMG cell over *N* = 8 fields per well × 3 wells per line and *N* = 2 separate SARS-CoV-2-exposed CB-IMG line derivations. Error bars represent SEM.

To illustrate the application of these models to study maternal exposures, we generated CB-iMGs from umbilical cord blood of neonates from mothers who tested positive for SARS-CoV-2 (*n* = 2). As observed above for CB-iMGs derived from SARS-CoV-2 unexposed pregnancies, these cells also display ramified and amoeboid morphology by phase contrast imaging (Fig. 4a), express microglial-specific markers IBA1, CX3CR1, PU.1, and P2RY12 by immunostaining (Fig. 4b, i–iv), and engulf pHrodo-labeled synaptosomes in our synaptic pruning assay as visualized by real-time live imaging (Fig. 5a–c) and quantification (Fig. 5d). These results demonstrate the capacity to create phenotypically characteristic and functionally active CB-iMGs in a quantitative model of synaptic pruning to provide for further investigations into potential effects of maternal exposures such as SARS-CoV-2.

## Discussion

Our results demonstrate the successful creation of neonatal patient-specific models of microglia-mediated synaptic pruning via cellular reprogramming of neonatal cord blood mononuclear cells. These models should facilitate novel insights into fetal brain development in the setting of maternal exposures, including but not limited to SARS-CoV-2 infection. Both SARS-CoV-2-exposed and -unexposed umbilical cord blood-derived microglia-like cells express canonical microglial markers IBA1, CX3CR1, PU.1, and P2RY12, and demonstrate a range of morphologies with varying degrees of ramification, potentially reflecting a range of activation states that can be perturbed in experimental systems. Importantly, the induced microglia phagocytose synaptosomes demonstrating that they can recapitulate this key function of microglia in the developing brain. This work suggests the potential for umbilical blood mononuclear cells to serve as a non-invasive, personalized biomarker of fetal brain microglial priming. To our knowledge, microglia have not previously been modeled from umbilical cord blood or used to predict neurodevelopmental vulnerability at a time when there is a window for intervention. These models provide the potential for quantifiable endpoints that can be used to assess microglial programming in the setting of various maternal exposures, and later can be used to test the efficacy of potential therapies to ameliorate in utero priming of microglia toward a pro-inflammatory phenotype^38^.

Previous studies have demonstrated the impact of various maternal exposures, including maternal stress, metabolic disorders, air pollution, and infections, on fetal brain development^2–4,7^. Such exposures may lead to offspring neurodevelopmental morbidity via maternal immune activation^39–46^, which results in aberrant microglial programming in the developing brain^12–15,18^. In turn, maternal immune activation models have pointed to aberrant differentiation of fetal microglia and dysregulation of cytokine networks as key mechanisms underlying abnormal fetal brain development, with microglia primed toward a pro-inflammatory phenotype and altered synaptic pruning implicated in offspring morbidity^11–15^. Given the extent of synapse formation and pruning that occurs in fetal and neonatal life, developmental microglial function represents a critical target for investigation.

We have previously demonstrated feasibility for the concept of using other monocyte populations to model microglial behavior using another monocyte type, fetal placental macrophages or Hofbauer cells, as a potential biologic surrogate for fetal microglial function in pre-clinical models of maternal obesity^16^, and have shown that maternal obesity-associated inflammation primes both fetal brain microglia and resident placental macrophages toward a highly correlated pro-inflammatory phenotype^16^. Personalized assays using more readily available cord blood mononuclear cells, as presented here, can be used to assess both baseline microglial function, and behavior in response to “second hit” inflammatory stimuli, which will be helpful in informing risk assessments for an individual fetus.

The COVID-19 pandemic, with its associated maternal immune activation and pro-inflammatory cytokine-mediated physiology^47,48^, may pose risk to the developing fetal brain. While current data suggest that vertical transmission of SARS-CoV-2 is relatively rare^49–51^, the profound immune activation observed in a subset of infected individuals suggests that, even if the virus itself does not cross placenta, the developing fetal brain may be impacted by maternal inflammation and altered cytokine expression during key developmental windows^52–54^. In this work, we suggest a model system that may be applied to investigate risk associated with the COVID-19 pandemic. Extending our work with adult peripheral blood mononuclear cells to cord blood-derived microglial models will allow for rapid, scalable models to investigate risk, yielding non-invasive, personalized assays of the impact of SARS-CoV-2 on fetal brain microglial priming and synaptic pruning function. This approach can complement more traditional approaches that will require large longitudinal cohort studies and may require years or even decades (in the case of schizophrenia, for example) to fully capture risk. The ability to detect priming of fetal brain microglia toward a pro-inflammatory phenotype extends beyond SARS-CoV-2 to include numerous other maternal infections in pregnancy, as well as the myriad maternal exposures that have been suggested to impact fetal microglial development.

In sum, we demonstrate the potential for umbilical cord blood mononuclear cells to serve as a non-invasive, personalized model of fetal brain microglial priming. These models provide the potential for quantifiable endpoints that can be used to assess microglial programming in the setting of various maternal exposures. We determined that CB-iMGs can recapitulate microglial characteristics and function in vitro, providing key insight into cells from the neonatal brain that are otherwise inaccessible at birth and throughout childhood. We illustrated their application to investigate effects of maternal infection, including SARS-CoV-2, on the developing brain^28^. Beyond characterizing any consequent abnormalities, the scalability of this approach may enable investigation of targeted therapeutic strategies to rescue such dysfunction.

## Supplementary information

Supplemental Material: Description of CellProfiler image analysis
