# Supplementary material for: Umbilical cord blood-derived microglia-like cells to model COVID-19 exposure
Source: Transl Psychiatry. 2021 Mar 19;11:179. doi: 10.1038/s41398-021-01287-w (PMC7976669; doi:10.1038/s41398-021-01287-w)
Supplement: Supplementary file 1 — Supplemental Material: Description of CellProfiler image analysis [file 41398_2021_1287_MOESM1_ESM.docx]

CellProfiler image analysis pipeline description

CellProfiler (version 3.1.9)^1^ was used to measure cell counts and synaptosome area using phase-contrast and red fluorescence live-cell images, respectively. Baseline phase-contrast images were processed using the EnhanceOrSuppressFeatures module. RobustBackground method thresholding was applied to processed images and used to identify cells based on size using the IdentifyPrimaryObjects module. To localize iMGs, phase-contrast images were enhanced with the EnhanceOrSuppressFeatures module. Then IdentifyPrimaryObjects segmented individual cells based on size and the thresholding method Robust Background. Measurements were collected using the module MeasureObjectSizeShape and exported to a spreadsheet.

Next we quantified the number and area of synaptosomes present in cells using a similar pipeline applied to the red fluorescence channel images. First, the GaussianFilter module was used to reduce noise. The images were then thresholded using the RobustBackground method in the Threshold module. Synaptosomes were segmented with the IdentifyPrimaryObjects module, which was set to segment based on both size and the Minimum Cross-Entropy thresholding method. The MeasureObjectSizeShape module recorded size metrics for the synaptosomes, which were then exported to a spreadsheet. Finally, the module OverlayOutlines was used in both pipelines to manually quality check the program’s segmentation of cells and synaptosomes.

For quantification of iMG conversion efficiency, we calculated the percentage of positively stained cells out of the total number of nuclei for each image. The IdentifyPrimaryObjects module was used to identify nuclei and areas of fluorescence (i.e., positive marker staining) within cells. Next, the RelateObjects module was used to relate fluorescence marker staining (child) to each nucleus (parent). The number of fluorescent objects overlaying with nuclei in an image (i.e., representing positively stained cells) was then divided by the total number of nuclei for the same image to plot the percentage (%) of positive cells per image, from either the adult or cord-blood derived iMG groups.

1. McQuin C, Goodman A, Chernyshev V, Kamentsky L, Cimini BA, Karhohs KW *et al.* CellProfiler 3.0: Next-generation image processing for biology. *PLoS Biol* 2018; **16**(7)**:** e2005970.
